# Effects of Estrogens on Osteoimmunology: A Role in Bone Metastasis

**DOI:** 10.3389/fimmu.2022.899104

**Published:** 2022-05-23

**Authors:** Julien C. Marie, Edith Bonnelye

**Affiliations:** ^1^ Cancer Research Center of Lyon (CRCL), Tumor Escape Resistance Immunity Department, INSERM-1052, CNRS 5286, Centre Léon Bérard, Université Claude Bernard Lyon 1, Lyon, France; ^2^ Univ. Lille, CNRS, Inserm, CHU Lille, Institut Pasteur de Lille, UMR9020-UMR1277-Canther-Cancer Heterogeneity, Plasticity and Resistance to Therapies, Lille, France

**Keywords:** estrogen, bone marrow, immune cells, bone metastasis, estrogen receptor

## Abstract

Bone loss associated with estrogen deficiency indicates a fundamental role of these hormones in skeletal growth and bone remodeling. In the last decades, growing recent evidence demonstrated that estrogens can also affect the immune compartment of the bone. In this review, we summarize the impacts of estrogens on bone immune cells and their consequences on bone homeostasis, metastasis settlement into the bone and tumor progression. We also addressed the role of an orphan nuclear receptor ERRalpha (“Estrogen-receptor Related Receptor alpha”) on macrophages and T lymphocytes, and as an immunomodulator in bone metastases. Hence, this review links estrogens to bone immune cells in osteo-oncology.

## Introduction

Estrogens family of steroid hormones composed of estradiol, estrone, estriol and estetrol. Estradiol (E2) is the most prevalent and the most potent estrogen. Though ovaries are the main producers of estrogens, fat tissues, testes, the adrenal cortex, and the liver also contribute to their production (1). Estrogens are major players in both skeletal growth, particularly during puberty, and skeletal maintenance, including normal bone mineral density and trabecular bone mass, during adult life. Reduced estrogen levels at menopause for women and later in life for men lead to decreased bone density and microarchitecture deterioration resulting in a high risk of fracture (1).

Estrogens exert their actions through genotropic and non-genotropic signaling pathways. They can bind to two nuclear receptor isoforms, estrogen receptor-α (ERα) and ERβ which, once their ligand pocket is occupied, migrate to the nucleus and fulfil their roles as transcription factors by binding to dedicated DNA sequences (2). ERα can also activate kinases (MEK, ERK, JNK) and modulates several transcription factor activities (c-jun, Elk-1) (3). More recently, a G Protein-Coupled Estrogen Receptor (GPER1) has been discovered. Unlike ERα/β, GPER1 binds estrogens with lower affinity (4). In addition, based on a strong similarity with ERα DNA binding domain (68%) but a moderate similarity (36%) with the ligand pocket of ER, precluding estrogen binding, three orphan nuclear receptors referred to as Estrogen receptor-Related Receptors (ERRs) ERRα, ERRβ and ERRγ have been described (5). Despite the identification of putative ligands, many data support the fact that ERRα impinges on the estrogen signaling pathway in numerous tissues including the bone (6–8).

Skeletal metastases are frequent complications of many cancers of which prostate (PCa) and breast (BCa) cancers are the most frequent with a 73% and 68% incidence of bone metastases (BMet) (9). BMet development requires first cancer cell extravasation and homing to the bone marrow through interactions with endothelial cells and with osteoblasts (bone-forming cells). Once in the bone, cancer cells can then disrupt the osteoblast/osteoclast balance; in favor of osteoclasts (bone-resorbing cells) or osteoblasts inducing osteolysis or osteoblastic/mixed lesions. Aside from bone cells, immune cells in the bone marrow (BM) also strongly influence BMet (10). In pre-clinical models, estrogen deficiency (OVX) has been shown to fuel BMet, since the declining production of the sex steroid by the ovaries and inflammatory tone associated with estrogen deficiency modified the bone microenvironment, mainly the osteoclasts, in such a way that cancer cell anchorage, survival and osteolytic phenotype were stimulated (11). Conversely in clinical studies, incidence of BM disseminated tumor cells (DTC) was reported to be slightly higher in pre-menopausal women (32.7%) versus postmenopausal (29.5%), suggesting that the BM of post-menopausal women is less attractive to metastasis (12). Of note, all immune cells present in the bone express the ERs (13), and growing evidence suggests a role for estrogens in BMet through their action on bone immune cells. Moreover, regarding basic science, it is important to keep in mind that a large part of the cultures of hematopoietic cells are carried out with media containing phenol red, known to have estrogenic activity. This review aims at presenting our current knowledge and our own thoughts on the links between estrogen signaling in bone immune cells and their impact on metastatic cell homing and progression within the bone/BM microenvironment.

## Estrogen and Myeloid Cells

Aside from erythrocytes, myeloid cells, including mainly neutrophils, monocytes/macrophages and osteoclasts are the most abundant hematopoietic cells in the bone (14). All of these myeloid cells, the differentiation of which occurs in partially or completely in the BM, express not only ERα/β but also GPER1 (15). We will see that estrogens affect both their differentiation and function in the bone with consequences on BMet niche, cancer cell homing and progression in the bone.

### Neutrophils

**Figure 1:**
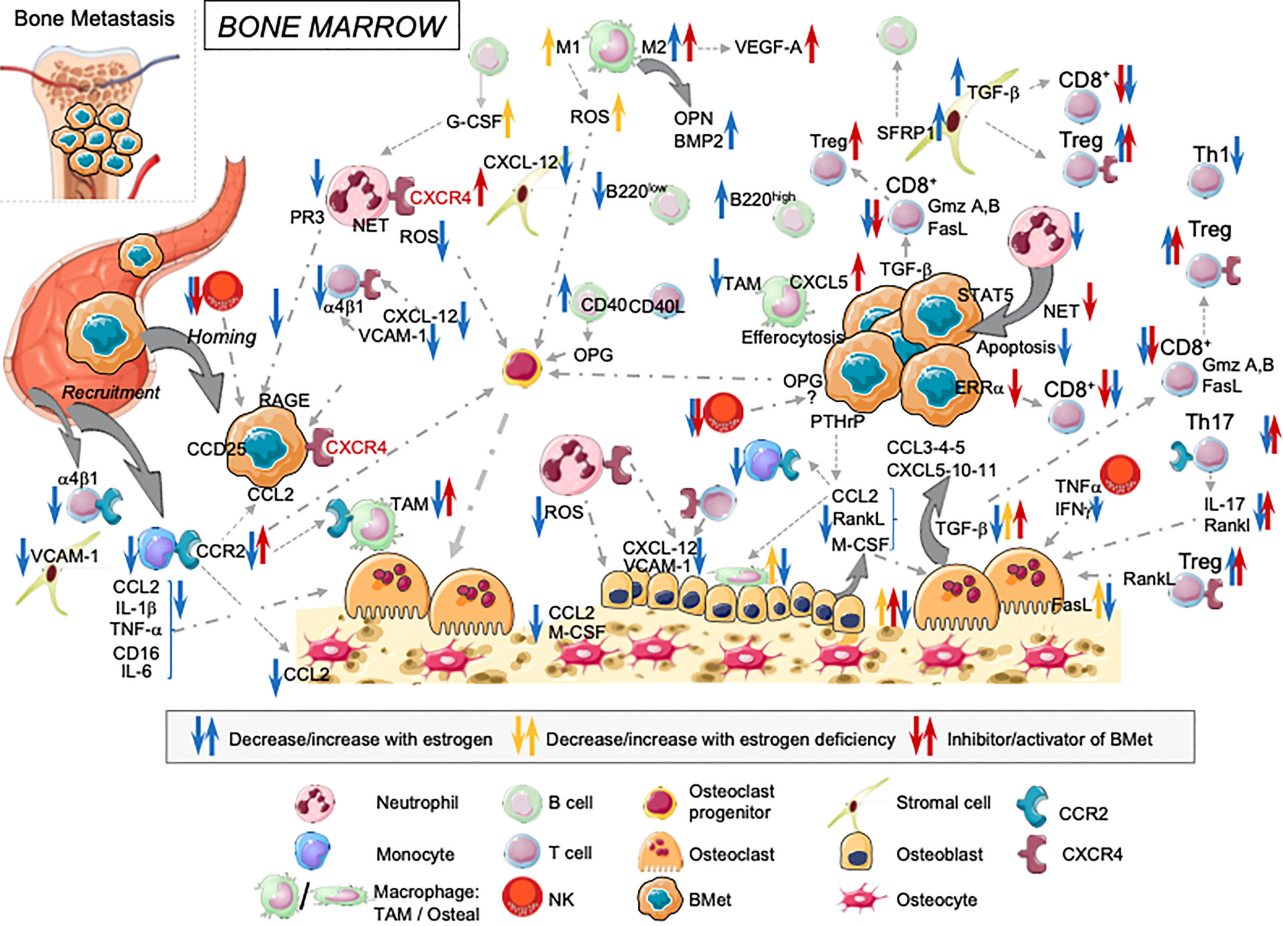
Overview of estrogens regulating immune landscape on the bone marrow cells: consequences on cancer cells anchorage and tumor progression in bone. Estrogens influence BM cells at different steps. They modulate both their development and functions with direct consequences on bone osteogenesis, cancer cell implantation and tumor growth in the bone.

Neutrophils largely outnumber the rest of the myeloid cells in the bone (16), where their numbers are influenced by estrogens. In the 80’s, experiments based on the injection of estrogens to male mice revealed a profound neutropenia in the BM (17, 18). Since then, it has been reported that estrogens impact both neutrophil differentiation and functions. Neutrophil differentiation is largely promoted by the Granulocyte-Colony-Stimulating Factor (GM-CSF). In the absence of estrogen, B cells present in the BM secrete more G-CSF, which can contribute to the neutrophilia observed in estrogen-deficient patients (19). Neutropenia in the bone associated with estrogens can also be explained by the ability of estrogens to down-regulate the production of CXC chemokine ligand (CXCL)12 by osteoblasts and BM stromal cells (20). Indeed, if a large portion of mature neutrophils leave the BM, the fraction expressing CXCR4, receptor of CXCL12, stays within the bone (20). These BM-resident neutrophils secrete Proteinase 3 (PR3), a serine protease which through its interaction with Receptor for Advanced Glycation Endproducts (RAGE) at the surface of metastatic PCa could enhance their homing to the bone (21). Though only the depletion of neutrophils *in vivo* will firmly validate this conclusion, this observation strongly suggests that a large number of neutrophils in the bone could contribute to BMet incidence from PCa cells by encouraging PCa cell anchorage in the BM. One feature of neutrophils is that they form NETs (Neutrophil Extracellular Traps), which not only prevents pathogen progression but also, as recently demonstrated, contributes to the sequestring of liver and lung metastatic cells in the bone promoting their implantation (22). Inhibition of ERα and ERβ signaling in neutrophils by using selective antagonist raloxifene was associated with inhibition of NETs suggesting that estrogen promote NETs formation into the bone (23). It is noteworthy that NET fails to attract primary cancer cells from breast to bone. This difference depending on metastases origin was associated with the ability of metastasis to express or not CCD25, which recognizes DNA chromatin filaments composing the NET (22). Once settled in the bone, BMets originating from PCa DTC stimulate oxidative bursts in neutrophils, increasing NET formation. In turn, neutrophils induce apoptosis of PCa cells by inhibiting STAT5 signaling in cancer cells in a manner that remains to be understood (24). Interestingly, this cytotoxic property of neutrophils decreases with time and in late-stage bone tumors. High levels of E2 also lead to a decrease in degranulation of β-glucuronidase and lysozyme, as well as in the intracellular concentration of reactive oxygen species (ROS) in neutrophils known to stimulate osteoclasts and osteoblast apoptosis (25–27). Hence, by controlling both neutrophil homeostasis and functions in the bone, estrogens repress the trapping of disseminated cells in the bone, but diminish the anti-tumor activity at an early stage and decrease the osteogenic feature associated with neutrophils once the tumor is settled within the bone (**Figure 1**).

### Monocytes-Macrophages

In addition to neutropenia, estrogens also repress the differentiation of mononuclear phagocytic cells. Indeed, estrogens reduce the levels of Macrophage-Colony-Stimulating Factors (M-CSF), mainly produced by osteocytes, osteoblasts, and osteoblast precursors as well as Granulocyte-Macrophage Colony-Stimulating Factor (GM-CSF), this latter being secreted by multiple cell types in the bone mainly in response to danger stimuli (28–31). Bone macrophages, mostly arise through the differentiation of monocytes returning to the bone. In addition to repressing monocyte/macrophage lineage differentiation, estrogens also reduce concentrations of CCL2 (C-C Motif Chemokine Ligand 2) produced by osteoblasts and osteocytes, and thus the recruitment to the bone of monocytes and macrophage-precursors which expressed CCR2 (32). Estrogens also directly inhibit the production of CCL2 by monocyte/macrophage and thus the positive loop of macrophage recruitment to the bone (31). Interestingly, depending on the ER engaged, estrogens can have different direct effects on monocyte/macrophage functions. In monocytes/macrophages, E2/ERα inhibits the production of pro-inflammatory cytokines, such as IL-1β, TNF-α, IL-6, documented to reduce osteoclast differentiation (31–33). Conversely, E2/ERβ represses CD16 surface expression and thus ADCC (antigen-dependent cellular cytotoxicity) (34). Whether different stages of macrophage maturation are more strongly associated with the expression of one type of ER than another remains to be addressed. ERRα, whose expression is up-regulated by estrogens in several tissues including the bone (8, 35, 36), is involved in macrophage functions. More precisely, it can regulate macrophage response to TLR4 and IFNγ as well as ROS production (37–39). Through their potent phagocytic activity, macrophages represent powerful cells to eliminate metastatic cells reaching the bone, albeit they can also influence BMet through other mechanisms that can be targeted by estrogens. “Indeed, estrogens strongly influence macrophage polarization by promoting M2 (pro-osteoblastic) and suppressing M1 (pro-osteoclastic) (34, 40). Therefore, by promoting M1 macrophages estrogen deficiency inhibits BMP2 production by M2 and stimulate pro-osteoclastic molecules production ROS, nitric oxide, and pro-inflammatory cytokines by M1, promoting bone resorption (41). If this balance in favor of M2 contributes to preventing the establishment of the metastatic niche in bone, once metastasis is implanted, M2-like macrophages promote metastasis angiogenesis and tumor progression through their production of VEGF-A (10). In the bone, a peculiar subset of macrophages named osteal macrophages, or osteomacs, has been described (42). Osteal macrophages (TRAP^-^ F4/80^+^, CD68^+^, Mac3^+^) are present at the bone surface in the close vicinity of mature osteoblasts where they support osteoblastic function and bone anabolism (43). Data have connected estrogen levels and osteal macrophage functions, since the number of osteal macrophages increased on both trabecular and endocortical bone post-ovariectomy. Interestingly, in this condition, osteal macrophages contain TRAP^+^ intracellular vesicles, attesting to their “clean up” role of exocytosed post-resorption vesicles released by osteoclast (TRAP^+^) activity (44). PCa-derived BMet increases osteoblast functions through osteoblastic production of CCL2, which favors osteal macrophage positioning (45). Tumor-associated macrophages (TAM) have been proposed potentiate BCa and PCa BMet. Increased numbers of CD206^+^ M2-like macrophages have also been found in PCa BMet (46, 47). CCL2 expression by cancer cells promotes the recruitment of TAM expressing CCR2 to facilitate cancer cell anchorage in the bone (33). In the same line, the expression of Parathyroid Hormone-Related Protein (PTHRP) by BCa and PCa cells up-regulates the production of CCL2 in osteoblasts that contribute to macrophage recruitment to the bone, bone remodeling and BMet progression (48, 49). Moreover, the clearance of tumor apoptotic cells by efferocytosis promotes CXCL5 production by macrophages and an inflammatory bone microenvironment supporting BMet development (50). Thus, estrogens repress the number of monocytes/macrophages in the bone including osteal macrophages and affect their functions. Estrogens enhance their non-inflammatory cytokine production and repress bone remodeling, preventing formation of a fertile soil for BMet anchorage. However, once BMet manage to settle in the bone, estrogens provide through their action on macrophages a microenvironment facilitating tumor progression (**Figure 1**).

### Osteoclasts

Osteoclasts are also derived from the monocyte/macrophage lineage. However, on inflammatory conditions, osteoclasts can arise from either dendritic cells or erythromyeloid progenitors (51–54). In addition to the indirect effects described above of estrogens on osteoclast lineage differentiation, selective ablation of ERα in osteoclasts was reported to induce trabecular bone loss in female mice due to decreased FasL expression and autocrine regulation, leading to the inhibition of osteoclast apoptosis (55). As for macrophages/monocytes, osteoclasts are also antigen-presenting cells able to activate CD4^+^T cells and CD8^+^ T cells, and are endowed with a unique ability to induce Foxp3^+^ regulatory T cells (Treg) (56, 57). Moreover, osteoclasts produce a number of chemokines (Cc13, Cc14, Ccl5, Cxcl5, Cxl10, Cxcl11) attracting multiple immune cells *e.g.* monocytes-macrophages, T-cells, NK and DC cells (58). PCa-derived BMet increase osteoblast functions and facilitate their growth in bone by activating osteoclastogenesis through osteoblastic production of the Receptor Activator of Nuclear factor Kappa-B ligand (RANKL), a key factor for osteoclast differentiation (45). ERRα is also a strong regulator of osteoclast differentiation (59–61). Interestingly, depending on the metastasis origin, overexpression of ERRα in cancer cells either inhibits or stimulates osteoclasts by stimulating the expression of either the decoy receptor of RANKL, OPG (osteoprotegerin), a major inhibitor of osteoclast activity in BCa cells, or VEGF-A and WNT5a in PCa cells (62, 63). Now the impact of estrogens on ERRα in oncology and their role on BMet modulation remains to be determined. Through their action on osteoclasts, estrogens repress bone resorption but also modify the ability of osteoclasts to interact and recruit other immune cells to the bone with direct consequences on the development of a pro-inflammatory microenvironment in favor of BMet (**Figure 1**).

## Estrogen and Lymphoid Cells

### T Cells

T lymphocytes present in the BM account for less than 5% of CD45^+^ cells and have a reduced CD4/CD8 ratio compared to the blood. The BM T cell compartment is almost exclusively composed of α,β T lymphocytes (64). A large part of the T cells present in the BM are memory cells, which either circulate or permanently inhabit the BM, suggesting that in the context of BMet, BM T cells could compose a pool of T cells with specificity against antigens bared by cancer cells (65). The entry of T cells into the BM is largely supported by CXCL12 produced by osteoblasts and stromal cells, and down-regulated by estrogens (20). In the absence of intravital/*in situ* labeling data, there is so far no direct evidence that estrogens affect T cell retention within the bone. However, this phenomenon is largely dictated by the interaction between α4β1 integrin, present at the surface of T cells, and VCAM-1 expressed by stromal cells and endothelial cells and depicted to be down-regulated by estrogens (66, 67). One of the main roles of estrogens on BM T cells is to repress their osteoclastogenic ability. Evidence of this function emerged more than 20 years ago, after the observation that in contrast to wild type mice, nude mice are protected from trabecular bone loss induced by ovariectomy (68, 69). Later, CD4 T cells producing IL-17 and RANK-L in the bone were identified as potent stimulators of osteoclastogenesis (70). More recently a Th17 osteoclastogenic T cell subset expressing high amounts of TNF-α, and established in the gut, was proposed to reach the bone in both mice and humans (71, 72), in a CCL2-dependent manner (73). Thanks to their osteoclastogenic ability Th17 cells have been suggested to establish a bone pre-metastatic niche, which facilitates cancer cell implantation in the bone (74) (75). In addition, Th17 cells were suggested to preclude BMet control anti-PD1 treatment (76). Estrogens directly control the Th17 cell pool since ERα binds the promoter of RORc inhibiting Rorgt expression (77, 78).

In addition to Th17 cell differentiation, estrogens repress the Th1 cell program (79). In the BM, estrogen deficiency impairs the production of TGF-β by stromal cells, a cytokine that plays a key role in the repression of T-Bet and IFN-γ/TNF-α expression in T cells (80) and thus in osteoclastogenesis (81). Given the potent role of TGF-β in repressing the expression of granzyme-A,-B, and FasL in CD8^+^ T cells (82), the ability of estrogens to sustain TGF-β levels in the BM impairs CD8^+^ T cell cytotoxic functions, and promotes BMet progression (83). However, the control by estrogens/estrogen signaling of TGF-β levels in bone and in BMet is more complex. Indeed, bone contains a large source of TGF-β stored in its mineralized matrix, which is released and activated by osteoclast activity. In addition, BMet can also contribute to increasing the levels of TGF-β (84). ERRα is also clearly involved in effector T cell activation (85, 86). In BCa BMet, ERRα expression restrains TGF-β production by cancer cells, leading to exacerbated cytotoxic features in CD8^+^ T cells in the bone and efficient BMet control (87). TGF-β has been associated with an increase in the generation and stability of Tregs (88). In the BM, the proportion of Tregs among CD4^+^ T cells is much higher than in the lymph nodes, likely due to high levels of CXCR4 expression on Tregs (89) largely promoted by estrogens (90). Once the cancer cell is anchored, Tregs produce high amounts of RANKL promoting osteolysis associated with a feedback loop of TGF-β release in BCa BMet (91).

Hence, in the bone, the effects of estrogens on T cells are dual: by contributing to an immune-suppressive environment associated with reduced activity of CD8^+^ T cells and increased number of Tregs which can favor tumor growth and by limiting osteolysis due to inhibition of RANKL production by Th1 and Th17 cells (**Figure 1**).

### B Cells

BM is the main site of B lymphopoiesis and maturation. In mice, estrogen treatment was linked to a decrease in the numbers of pro/pre- (B220^low^/IgM^−^) and immature (B220^low^/IgM^+)^ B subpopulations, whereas the mature (B220^high^/IgM^+^) subpopulation increased in the BM (92). This modification of B cell precursor homeostasis does not seems to be associated with a loss of survival of pro/pre- and immature B cells since estrogens induce an increase in the anti-apoptotic factor Bcl2 in these cells (93). The exact mechanism of action of estrogens on B lymphopoiesis *in vivo* remains unknown. However, co-culture approaches suggested that estrogens modify the ability of BM stromal cells to support efficient B lymphopoiesis (94). Estrogens promote the production of soluble Frizzled-related protein 1 by BM stromal cells, which in turn stabilizes β-catenin and blocks early B lymphoid progression (95). Of note, B cell interactions with other immune cells in the BM conditions bone homeostasis. Through CD40/CD40L interaction, B cells and T cells cooperate to sustain normal bone mass and mineral density. Signals delivered by CD40 engagement stimulates in B cells the production of OPG and consequently reduce osteoclast differentiation. Interestingly, in post-menopausal women, a switch from OPG to RANK-L production is observed in B cells emphasizing bone loss (96, 97). The role of B cells in the direct control of BMet seems elusive, since in mice with BMet, as in humans, mature B cells are scattered over the BM close to osteoclasts rather than metastasis (98). However, by controlling B lymphopoiesis, and modifying mature B cell profile in the BM, estrogen levels modify bone homeostasis by reducing osteolysis and thus may limit cancer cell anchorage and progression in the bone.

### Innate Lymphoid Cells (ILCs)

In the adult, the BM is regarded as the main site for ILC-poiesis. Several multipotent ILC precursors have been defined in mice with different degrees of pluripotency that are still under debate (99). Among ILCs, ILC-1/Natural killer (NK) cells express both ERα/β and are prevalent in the BM. In contrast, outside the uterus ILC-2 fails to express ERs, and ILC-3 are endowed with a clear tropism for the mucosa (100). If the effects of estrogen on NK cell ontogeny in the BM are not well documented, there is more evidence of a role, either direct or indirect, for estrogens on NK functions in BM. Interestingly, both development and activation of BM NK cells are dependent on IL-15, a cytokine also important for osteoclast development (101) and abundantly produced by a fraction of mesenchymal cells named CAR cells (CXCL12-abundant reticular) (102, 103), the functions of which are orchestrated by estrogens. Once NK cells have matured, the down-regulation of CXCR4 and up-regulation of S1P5 drive their egress from the CXCL12 enriched BM microenvironment (104). A large population of resident CXCR6^+^CD69^+^ NK cells is observed but seems to be endowed with a weak cytotoxic ability against cancer cells, whereas the major anti-tumor activity of NK is supported by recirculating mature NK cells (105). Estrogens reduce the cytotoxic activity (production of granzymes and FasL) of mature NK cells, likely through the down-regulation of their activation receptors (NKp46, NKG2D/L) and the increased expression of the inhibiting receptor CD94 (106). However in the bone, no effect of NK on BMet progression has clearly been documented (107). Hence, the reduced cytotoxic function of NK cells in response to estrogens likely increases the number of disseminated cells in the blood that could reach the bone, but do not contribute to the metastasis immune escape once settled in the bone. However activated NK cells can either promote or inhibit osteoclastogenesis depending on the release of TNF-α or IFN-γ, respectively and this regulation is influenced by estrogens which repress IFNγ in NK cells (106, 108, 109). Activated NK cells can also directly lyse mature osteoclasts (110). Conversely, zoledronate treatment, the most potent bisphosphonate for the treatment of BMet, protects osteoclasts from NK cell cytotoxicity (110). Thus, estrogens induce a large repression of NK cell functions in the bone, which in the light of the ability of NK cells to regulate bone resorbing cells could impact bone remodeling and BMet progression (**Figure 1**).

## Conclusion

The last decades of works on estrogens and BM brought to light the fact that estrogens affect the immune compartment of the bone and possibly the global system. Therefore, this observation may explain why adult females mount stronger immune responses than males, and strongly questions the consequences of modifications in estrogen levels on innate and adaptive immune responses during aging and gender reassignment. Moreover, the emergence of the immunomodulatory role of estrogens suggests that anti-estrogenic drugs, or estrogen administration, by injection or topical applications, may influence the BM. Subsequently this treatment may have an indirect anti-tumor effects by modulating the immune system and may therefore participate to the modulation of pre-metastatic niches and/or modulate the tumor immune environment in the bone. Anti-estrogenic/and estrogenic therapies may also modify the ability of patients to respond to immunotherapy by affecting BM immune cell development and function. This area needs to be particularly explored given the increasing number of people either treated with anti-estrogenic drugs or receiving estrogen.

## Author Contributions

JM and EB contributed equally to this work. All authors contributed to the article and approved the submitted version.

## Conflict of Interest

The authors declare that the research was conducted in the absence of any commercial or financial relationships that could be construed as a potential conflict of interest.

## Publisher’s Note

All claims expressed in this article are solely those of the authors and do not necessarily represent those of their affiliated organizations, or those of the publisher, the editors and the reviewers. Any product that may be evaluated in this article, or claim that may be made by its manufacturer, is not guaranteed or endorsed by the publisher.

## Funding

This work was supported by the National Center for Scientific Research (CNRS) to EB, the National Institute of Health and Medical Research (INSERM) to JM, by the Labex DevWECan ANR-10-LABX-6 (JM). The SIRIC LYriCAN INCa-DGOS-inserm_12563 is acknowledged.
